# Incidence of occupational injuries among adults residing in a selected rural area of India: A cross sectional study

**DOI:** 10.12669/pjms.35.3.293

**Published:** 2019

**Authors:** Shilpa Ravi, Bobby Joseph

**Affiliations:** 1 *Dr. Shilpa Ravi, MD, DNB. Centre Physician In charge, Healthspring Family Health Experts, Mumbai, India*; 2 *Prof. Dr. Bobby Joseph, MD, DNB. Department of Community Health, St. John’s Medical College, Bangalore, India*

**Keywords:** Agriculture, Injury, Rural occupations, Incidence

## Abstract

**Objectives:**

The objectives of this study were to assess the incidence and profile of occupational injuries among rural workers of rural India.

**Methods:**

This study was conducted among all persons between the ages of 18 to 60 years and engaged in some occupation and residing in the villages under the three rural subcentres of Sarjapur Primary Health Centre during the time period of 2009-2012. The sample size was calculated to be 400 assuming a prevalence of 10% and absolute precision of 3% at 95% confidence limits and a systematic random sampling of the household was done to select the study population.

**Results:**

The incidence of occupational injuries in the study period of 2009-2012 was 22%. Out of the total 91 injuries, the proportion of injuries, which were agriculture-related, was 62%. The most common cause of injury was due to contact with plant thorns. Above half (54%) involved the upper limbs, and (30%) involved the lower limbs. By using the injury severity scale, 88% were of minor degree. 67% required treatment and 32% of the injured persons took treatment in a private hospital. Those who sustained an injury, 9% required admission to a hospital. Three participants have sustained a permanent disability during this study.

**Conclusion:**

The Incidence of occupational injuries was found to be 22% and agriculture showed to have the highest proportion of injuries.

## INTRODUCTION

There are many occupations in the world. An individual spends a third of his adult life working and each occupation carries its own characteristic hazards.^1^ The Census of India document 2001^2^ defines work as participation in any economically productive activity with or without compensation, wages or profit.

According to ILO estimates for the year 2018, 62.6% of the working population in low income countries are employed in Agriculture and 43.9% of Indians are employed in Agriculture.^3^

The active work-force comprises approximately 630 million employed work population in the South East Asian Region. Agriculture is the major sector providing employment to 65% of active workforce in the region and in India 58% of the population is involved in agriculture.^4^ Even though there is at present a trend toward urbanization, most of the population lives and works in rural areas and International Labour Organisation estimates that 1.1 billion workers are engaged in agriculture.^5^ Since agriculture contributes to the major occupation in Rural India, the present study shows that majority of persons are involved in agriculture and so are the corresponding injuries.

Injuries are a major public health problem in India. Lack of reliable and good quality national or regional data has thwarted their recognition. Many injuries are linked to social, environmental, cultural and biological issues in causation and are recognised as man-made, behaviour-linked or due to socio-demographic transition.^6^

Occupational hazards cause or contribute to the premature death of millions of people worldwide and result in the ill health or disablement of hundreds of millions more each year. The burden of disease from selected occupational risk factors amounts to 1.5% of the global burden in terms of DALY (Disability adjusted life years) lost. The World Health Report 2002 places occupational risks as the tenth leading cause of morbidity and mortality.^7^

As per the International Labour Organization it is estimated that worldwide there are at least two million deaths per year due to occupational diseases and injuries. An estimate in India suggests that there are around 3.1 lakh work-related deaths per year.^8^

In the agricultural sector, the injuries occur more commonly as a result of cut due to knife while cutting plants. Where preventive programmes are not available or are not yet well developed, such injuries may well become infected. In addition, there is also the risk of tetanus.^9^ Though this study focusses on all the different occupations, since agriculture is the predominant occupation, the injuries found in agriculture are more in number and need intervention.

The objectives of this study were to assess the incidence of occupational injuries among rural workers in three rural subcentres of a Primary Health Centre in Bangalore District and to study the profile and factors leading to occupational injuries in the workers.

## METHODS

This cross-sectional study was undertaken in the villages under the three rural subcentres of Sarjapur Primary Health Centre, Bangalore District, Karnataka State, South India. All persons in the age group of 18 to 60 years involved in any occupation and residing in the villages coming under the three rural subcenters of Sarjapur Primary Health Centre formed the universe of the study. A systematic random sampling of the households was done within a household to select the study population. The sample size was calculated to be 400 assuming a prevalence of 10% and absolute precision of 3% at 95% confidence limits by using the formula of N=z^2^PQ/D^2^. The survey was started using the sampling interval that was calculated based on the population size and sample size and every 9^th^ household was surveyed.

During data collection it was found that there were more houses than the number provided by the Primary Health Centre. On completion of the systematic random sampling, a total of 409 houses were visited. Men and women in the age group of 18 to 60 years and those who were involved in any economically productive activity with or without compensation, wages or profit were included in the study. Injuries which had occurred on the way to or returning from work were not considered. According to Census 2001 all those who came in the category of non-workers (housewives, students, beggars, others, etc.) were excluded from the study population.

The definition of injury used in this study was similar to that used by the United States Bureau of Labour Statistics which defines occupational injury as “any injury such as a cut, fracture, sprain, amputation, etc., which results from a work-related event or from a single instantaneous exposure in the work environment”.^10^

The data was entered in Microsoft Excel spreadsheet and analysed using standard statistical software packages. Frequencies, percentages and chi-square test were used to study the associations between selected demographic variables and incidence of injuries.

## RESULTS

A total of 409 persons were included in the study. Of the total 409 persons, 283 (69.2%) were males. A large majority of the study population were in the age group of 31-45 years (177, 43.3%). Of the study subjects, 241 (58.9%) were married and a large majority (355, 86.8%) belonged to Hindu religion. A large majority of the population fell into the illiterate category (159, 38.9%) and 194 (47.4%) of the study population belonged to high socio-economic status according to the standard of living index. A large majority of the rural study population were involved in agricultural activities (254, 62.1%). The study area was rapidly transitioning and had been influenced by urbanisation and industrialisation. This had led to people being employed in the non-agricultural sector. About 26.9% of the population were skilled manual labourers and 3.7% were unskilled manual labourers. (Table-I & Table-II)

**Table-I:** Age & gender distribution.

Age group (in years)	Gender		Total (%)

	Females (%)	Males (%)	
18 – 30	53 (38.1)	86 (61.9)	139 (34.0)
31 – 45	55 (31.1)	122 (68.9)	177 (43.3)
46 – 59	18 (19.4)	75 (80.6)	93 (22.7)
Total (%)	126 (30.8)	283 (69.2)	409 (100)

N = 409

**Table-II:** Occupational profile with the frequency of accidents.

Occupation	Frequency	Percent	No. of accidents
Professional, technical, managerial	13	3.2	1
Clerical	2	0.5	0
Sales	9	2.2	3
Agriculture – self employed	245	62.1	57
Services	6	1.5	1
Skilled manual	110	26.9	27
Unskilled manual	15	3.7	2
Total	409	100.0	91

N = 91

Ninety-one persons sustained an occupational injury in the last one year. Therefore the incidence of occupational injuries was 22.25%. (222.5 per 1000 adult workers). The incidence of agricultural injuries was 13.94% (139.4 per 1000). Of the total 91 injuries, the proportion of injuries which were agriculture related was 62.64%. Of the non-agricultural injuries, a majority had taken place among women who work in garment manufacturing factories in the vicinity of their residence.

The mean age of the population among those who sustained an injury was found to be 37.9 years with a SD of 11.9 years. Of the 91 persons who had suffered from an injury in the past one year, 39 (42.9%) had acquired the injury in the last one to six months. A large majority of agricultural injuries had occurred in the harvesting season. Of the total number of persons who sustained injuries, about 34 (37.4%) occurred in the monsoon season.

The most common cause of injury was due to contact with plant thorns (in the farms) followed by injury due to pricks by needles. This being a peri-urban region, the latter injury was common in persons working in the garment manufacturing industry. The most common body part involved in the injury was the upper limb (49, 53.8%) especially the forearm, hands and fingers. By using the Injury Severity Scale^11^, 80 (87.9%) were of minor degree and 10 (11%) were of moderate degree and only 1 (1.1%) of the injuries was severe. Of the total number of injuries sustained, 39 (42.9%) of the persons sustained a laceration, 34 (37.4%) had abrasions and 7 (7.7%) had contusion and fractures as the type of injury.

Of the 91 who sustained an injury, it was found that about 79 (86.8%) did not have any restriction of daily activities and a large majority (88, 96.7%) did not have any permanent disability. All the three persons who had a permanent disability were involved in agriculture.

Of the 91 persons who had sustained an occupational injury, 62 (68.1%) required some form of treatment. Twenty (32.26%) of the injured persons took treatment in a private hospital and more of the injuries were minor than major, requiring simple treatment and rarely requiring admission to a hospital. 8 (8.79%) required admission to a hospital who had sustained agricultural injuries.

Eighty eight (96.7%) found lighting to be satisfactory in the workplace, 53 (58.2%) of the injuries were sustained in the afternoon and 56 (61.5%) of the study population felt that the temperature of the environment to be normal. 20 (35.1%) occurred in the monsoon months of the year. In general, climatic conditions had no role in the injuries.

Nine persons were suffering from a chronic illness and five out of them were on long term medication. Four injuries could have been prevented by using footwear as a form of personal protective equipment. On enquiring about the number of hours worked prior to injury, a large proportion of injuries (55, 60.4%) occurred between 8 to 12 hours after working. Financial burden was experienced by 19 (21%) of the individuals who sustained an injury. Three out of the 91 persons who sustained an injury had to completely stop working due to a permanent disability. (Table-III)

**Table-III:** Profile of occupational injuries.

Injury related			

Cause of injury	Number (%)	Body part involved	Number (%)
Contact with plant thorns	26 (28.7)	Hands/ fingers	49 (53.9)
Contact with needle	18 (19.8)	Legs	27 (29.7)
Contact with knife, sword or dagger	14 (15.4)	Feet/ankle/toes	9 (9.9)
Struck by thrown, projected or falling object	13 (14.3)	Chest/ back	4 (4.4)
Striking against or struck by other object	12 (13.2)	Others	2 (2.1)
Others	8 (8.7)	Total	91(100)

*Severity (ISS)*	*Number (%)*	*Type of injury*	*Number (%)*

Minor	80 (87.9)	Laceration	39 (42.8)
Moderate	10(11.0)	Abrasion	34 (37.4)
Severe	1 (1.1)	Others	18 (19.8)
***Individual related***			
Presence of acute illness	1 (1.1)	Presence of chronic illness	9 (9.9)
Use of alcohol 24 hours prior to the injury	1 (1.1)	On long term Medication	5 (5.5)
***Effects of the injury***			
Presence of financial burden	19 (20.0)	Presence of social Burden	1 (1.1)
Presence of family burden	11 (12.1)	Presence of psychological burden	1 (1.1)
Permanent disability	3 (3.3)	More than 30 work loss days	10 (11.0)

N=91

## DISCUSSION

This study has tried to assess the incidence of occupational injuries in a rural area. The study also focuses on the determinants and factors which lead to the occurrence of injuries at the workplace. In the present study, out of the total 409 persons, 69% were males. The reason for this skewed distribution in gender was due to the fact that females who were mainly housewives were excluded from the study. In a population based study done in Ontario, high injury rates were observed in the males, which were similar to the results obtained in the present study.^12^ In the present study, a large majority of the study population were in the age group of 31-45 years (43%) which was similar to the findings of the above mentioned study which showed high injury rates in the 31-40 years age. This indicates that injuries are common in the economically productive age group.

The annual incidence of injuries in all occupations was 222.49 per 1000 working adults. The study area was a peri-urban area influenced by urbanisation and industrialization and this had led to people being employed in non-agricultural sector and therefore had an influence on the incidence and profile of injuries. In a study done in farmers in Denmark, during the 12-month period, among farm owners, 35% experienced at least one injury per year.^13^ This higher incidence of occupational injuries which was probably due to the fact that it was a prospective study which was done with a one year follow up based on weekly registration. This could have resulted in a better recall and thereby better reporting and description of the injuries.

. In a study done in North Eastern France in 2004, the annual incidence rate of at least one occupational injury was 4%. This study included the different occupations similar to the present study. The incidence of injuries was lower probably due to the reason that a mailed questionnaire was used to collect the data. This could have lead to more chance of dropout.^14^

In the present study, 42% had acquired the injury in the last one to six months and this corresponded to an increased incidence of injuries in the harvesting season. The reason for the higher incidence of injuries in the harvesting season is probably due to increased working hours during that time which leads to fatigue and therefore injuries in the workplace. The other reasons could be increased quantity of work and more exposure to the sun. In a study done to assess agricultural accidents in 1969 in Cambridge, there was a peak in the number of accidents in late summer and autumn, which coincided with the harvesting season. The findings were consistent with the findings in the present study which also showed a higher incidence of injuries in the harvesting season.^15^

In a population based study done in Ontario to assess non-fatal injuries, the common mechanisms of injury included injuries related to the use of farm machinery, overexertion from lifting, accidental falls, and injuries that occurred while working with farm animals.^12^ In the present study, the most common cause of injury was contact with plant thorns. Most of the agriculture was not mechanised and manual work was predominant. In a study done in the year 1969 in Cambridge, out of the total 132 individuals who sustained an agricultural accident, 17 of them had a permanent disability. Thereby the prevalence of disability was 12% as compared to our study which showed prevalence of 3%. The reason for this difference could be that the Cambridge study was done in patients in a hospital. Therefore more number of persons who had a severe injury and thereby disability visited the hospital for treatment.^15^

Of the 91 who sustained an injury, one person had consumed alcohol 24 hours prior to the injury. A large majority of 87 (95%) did not remember if they had consumed alcohol 24 hours prior to sustaining the injury. The reason for asking this was because injuries are more common when a person is under the influence of alcohol. There are various reasons for consumption of alcohol like job dissatisfaction due to lack of pay, financial and domestic problems. This can lead to lack of proper attention and concentration in the job and thereby injuries in the workplace.

Financial burden was experienced by 19 (20%) of the individuals who sustained an injury due to a permanent disability and injuries which had resulted in hospitalisation for a longer duration of time leading to loss of pay or daily wages due to absence from work and due to the money which was spent on treatment and hospitalization. The same reasons could be attributed to the burden faced by the family members as a result of loss of family income. Social and psychological burden was faced by those who had a permanent disability as a result of injury.

Four injuries could have been prevented by using footwear as a form of personal protective equipment. The injuries were due to being hit by a projecting or falling object like a piece of wood on the ground. In a study published in 2002 on the assessment of PPE use among Mid Western farmers in the United States of America, the PPE usage was found to be low. Farmers were satisfied with availability of PPE through local hardware and farm cooperatives, but the decision to use PPE was personal and influenced little by outside parties.^16^ This is similar to the present study where the usage of PPE was low among those who sustained an injury. The reasons for poor usage could be non-availability of PPE, difficulty and cumbersomeness in use, lack of training, inability to purchase PPEs due to cost and lack of knowledge regarding importance of use of PPE during work.

In another study done in Malaysia to assess non-fatal occupational injuries from 2002-06, the agriculture sector reported the highest incidence rate (24.1/1,000), men aged 40 to 59 years and persons of Indian ethnicity had a greater tendency to sustain injuries.^17^ In the present study, the rate of occupational injuries was 222.25 per 1000 workers with increased incidence of injuries reported by the agricultural sector (23%). The reason for lower incidence of injuries in the Malaysian study could be due to better training of workers and pre-placement examinations before recruitment. The other reason could be that this was a record review done based on injuries reported to the Malaysia’s Social Security Organisation and therefore minor injuries would have not been reported and missed. Also if the number of people reporting the injuries were from the same occupation, the incidence of injuries would be higher when compared to other occupations which do not report injuries adequately.

### Limitation of the study

The incidence of injuries was assessed with a one year recall period. This could have lead to reduced recollection of the injuries and the events which lead to the injuries and thereby a lower incidence was obtained

### Recommendations

In order to prevent agricultural injuries, the persons involved in agriculture must be educated about injuries, the cause and mechanism and the modes of prevention. The use of personal protective equipment is an important component in prevention. All workers should receive education and training in the tools they are to use and the way in which they should be used. For hand safety, special gloves are available which can be used. Apart from the PPE to prevent hand injuries, use of footwear is also essential as many injuries occur due to pricks by thorns as a result of walking barefoot. The importance of what to do when an injury occurs in the workplace i.e. training of persons involved in agriculture on first aid should be stressed.

Given the likelihood of villagers finding gainful employment in nearby industries, attention should also be focussed on the various levels of prevention in these factories. For example, in the present study there were a number of cases of needle injuries among women working in the garment industry. Employing the principles of personal protection and safe machine handling can go a long way in reducing the burden of injuries among those in the rural region who opt for “better” opportunities in the industrial sector.

### Authors’ Contribution

**SR:** Conceived, designed, collected data, analysed data and prepared the manuscript.

**BJ:** Conceived, designed and reviewed and approved the manuscript.
